# Ursolic acid derivative UAOS-Na treats experimental autoimmune encephalomyelitis by immunoregulation and protecting myelin

**DOI:** 10.3389/fneur.2023.1269862

**Published:** 2023-11-30

**Authors:** Maolin Wang, Chenming Gu, Yifu Yang, Liang Chen, Kaixian Chen, Jun Du, Huali Wu, Yiming Li

**Affiliations:** ^1^School of Pharmacy, Shanghai University of Traditional Chinese Medicine, Shanghai, China; ^2^Department of Pharmacy, The Affiliated Hospital of Southwest Medical University, Luzhou, China; ^3^Experiment Center for Science and Technology, Shanghai University of Traditional Chinese Medicine, Shanghai, China; ^4^Nutrition Science, Amway (Shanghai) Innovation and Science Co., Ltd., Shanghai, China

**Keywords:** experimental autoimmune encephalomyelitis, ursolic acid, MHC-I antigen presentation pathway, TAPBP, H2-T23

## Abstract

**Introduction:**

Multiple sclerosis (MS) is an inflammatory demyelinating disease of the central nervous system (CNS). Ursolic acid (UA) can be used in the MS treatment with anti-inflammatory and neuroprotective activities. However, UA is insoluble in water, which may affect its medication effectiveness. In our previous study, UAOS-Na, a water-soluble derivative of UA was obtained. In this study, we evaluated the pharmacological effects and explored its underlying mechanism of UAOS-Na on experimental autoimmune encephalomyelitis (EAE).

**Methods:**

Firstly, the pharmacodynamics of UAOS-Na was investigated in EAE and Cuprizone-induced mice. And then the possible mechanisms were investigated by TMT proteomics and verified by in vitro and in vivo experiments.

**Results:**

UAOS-Na (30 mg/kg/d) delayed the onset time of EAE from 11.78 days post immunization (dpi) to 14.33 dpi, reduced the incidence from 90.0% to 42.9%. UAOS-Na (60 mg/kg/d) reduced the serum levels of IFN-γ, IL-17A, TNF-α and IL-6, reduced the mononuclear cell infiltration of spinal cord, and inhibited the overexpression of key transcription factors T-bet and ROR-γt of EAE mouse spinal cord. In addition, UAOS-Na attenuated demyelination and astrogliosis in the CNS of EAE and cuprizone-induced mice. Mechanistically, proteomics showed that 96 differential expression proteins (DEPs) were enriched and 94 were upregulated in EAE mice compared with normal group. After UAOS-Na treatment, 16 DEPs were enriched and 15 were downregulated, and these DEPs were markedly enriched in antigen processing and presentation (APP) signaling pathway. Moreover, UAOS-Na downregulated the protein levels of Tapbp and H2-T23 in MHC-I antigen presentation pathway and reduced the proliferation of splenic CD8 T cells, thereby inhibiting the CNS infiltration of CD8 T cells.

**Conclusion:**

Our findings demonstrated that UAOS-Na has both myelin protective and anti-inflammatory effects. And it could reduce the inflammation of MS by downregulating the expression of Tapbp and H2-T23 in the MHC-I antigen presentation pathway.

## Introduction

Multiple sclerosis (MS) is a common central nervous system (CNS) disease, which can lead to non-traumatic disability in young and middle-aged people (1). As a chronic neurodegenerative disease with a high disability rate, MS imposes a heavy burden on families and society (2, 3). MS pathology mainly includes inflammation and demyelination. Disease-modifying therapy (DMT), including nonspecific immunomodulators and targeted monoclonal antibodies, are the main clinical drugs for MS, but they cannot effectively suppress disease process and have serious adverse reactions (4, 5). For example, IFN-β and Glatiramer acetate inhibit the proliferation, differentiation, and antigen presentation of CD4 T cells, and restore the immunomodulatory function, but about 30% of relapsing remitting MS patients are not sensitive to them (6, 7). IFN-β causes influenza-like symptoms and rare hepatotoxicity (8). Fingolimod, a S1P1 antagonist, blocks the efflux of lymphocytes from lymph nodes, resulting in lymphopenia. Fingolimod only stops the outflow of lymphocytes without depleting them, so discontinuation of the drug can lead to disease outbreaks (9, 10). Alemtuzumab, an anti-CD52 monoclonal antibody, depletes immune cells by binding to CD52 on the surface of lymphocytes and monocytes. it is one of the most effective DMT in the treatment of MS, but it is associated with serious adverse events, including sepsis, secondary autoimmunity, stroke, and even death (11–13). In parallel, several neuroprotective drugs already used to treat other neurodegenerative diseases are currently undergoing clinical trials in MS, but the results are not promising. This prompts us to turn our attention to MS therapies with dual effects of immunomodulation and neuroregeneration.

According to statistics by 2019, natural products and their derivatives account for 23.5% of the drugs approved by FDA, indicating natural products are still an invaluable source to find lead compounds and pharmacophores (14). Ursolic acid (UA) is a natural pharmaceutical chemical component with various biological activities such as anti-inflammation (15), neuroprotection and promoting neuroregeneration (16, 17). UA has been reported to have dual effects, including inflammatory regulation by inhibiting protein expression in the NF-κB, STAT3/6, and Akt/mTOR signaling pathways (17–20), and directly remyelination in MS mice model by activating PPARγ signaling pathway and upregulating the expression of myelin-related genes (21–24). These previous studies supported UA as a potential treatment for MS.

It is well known that low aqueous solubility of a drug may seriously affect its medication effectiveness. UA is insoluble in water and has poor bioavailability in the humans (25, 26). To promote the aqueous solubility of UA, its derivative UAOS-Na was obtained by forming a C-3 hydroxyl ester with succinic acid and then alkalizing to a salt. In this study, the pharmacodynamics and mechanism of UAOS-Na in experimental autoimmune encephalomyelitis (EAE, an animal model of MS) treatment were explored. Compared to UA, we found that UAOS-Na was more effective including delaying the onset time and reducing the incidence of EAE. UAOS-Na also had a dual effect through reducing inflammation and protecting myelin of MS animal models. Mechanistically, UAOS-Na treatment downregulated the protein levels of Tapbp and H2-T23 in MHC-I antigen presentation pathway and reduced the proliferation of splenic CD8 T cells, thereby inhibiting the CNS infiltration of CD8 T cells.

## Materials and methods

### Animals

Healthy female/male C57BL/6 mice (17–20 g, 6–8 weeks old) were purchased from Vital River Laboratory Animal Technology Co., Beijing. China (certificate number: 20210520Abzz 0619000101). The animals were maintained in a specific-pathogen-free room at a temperature of 22°C ± 3°C and humidity of 55% ± 5% under a 12 h light/dark cycle. All animal experiments and protocols were conducted in strict accordance with the relevant regulations. The study protocol was approved by the Ethics Committee of Animal Experiments of Shanghai University of Traditional Chinese Medicine (approval number: PZSHUTCM 201023015).

### EAE induction

After 1 week of adaptive feeding, female C57BL/6 mice were immunized at three sites on the back (the middle of both shoulders and upper the groin on both sides) with 4 mg/mL heat-killed *Mycobacterium tuberculosis* H37Ra (#231141; BD, Franklin Lakes, NJ, United States) with 200 μg of myelin oligodendrocyte glycoprotein (MOG) 35-55 (#T510219-0001; Sangon Biotech, Shanghai, China) in 200 μL of emulsion containing 50% complete Freund’s adjuvant (#F5506; Sigma, St. Louis, MI, United States). The mice were then intraperitoneally injected with 400 ng pertussis toxin (#GC17532; GLPBIO, Montclair, CA, United States) in phosphate buffered saline (PBS) at 0 and 2 days post immunization (dpi). All animals were weighed and scored daily in a blinded manner using *Weaver’s 15-point scoring system* (27, 28). In terms of tail scores, a score of 0 indicated no clinical signs, a score of 1 indicated reduced tail tension, and a score of 2 indicated tail paralysis. In terms of limb scores, a score of 0 indicated no clinical signs, a score of 1 indicated waddling gait, a score of 2 indicated mild limb paralysis (limb dragging while walking), and a score of 3 indicated limb paralysis. The tail and limb scores were then accumulated, and a score of 15 represented death. For therapeutic administration experiments, mice were given individualized treatment, that is, mice were treated only after they developed clinical symptoms (onset, 10–14 dpi). Mice that did not onset until 15 dpi after modeling were not included in this study.

### Medicine preparation

**Figure 1:**
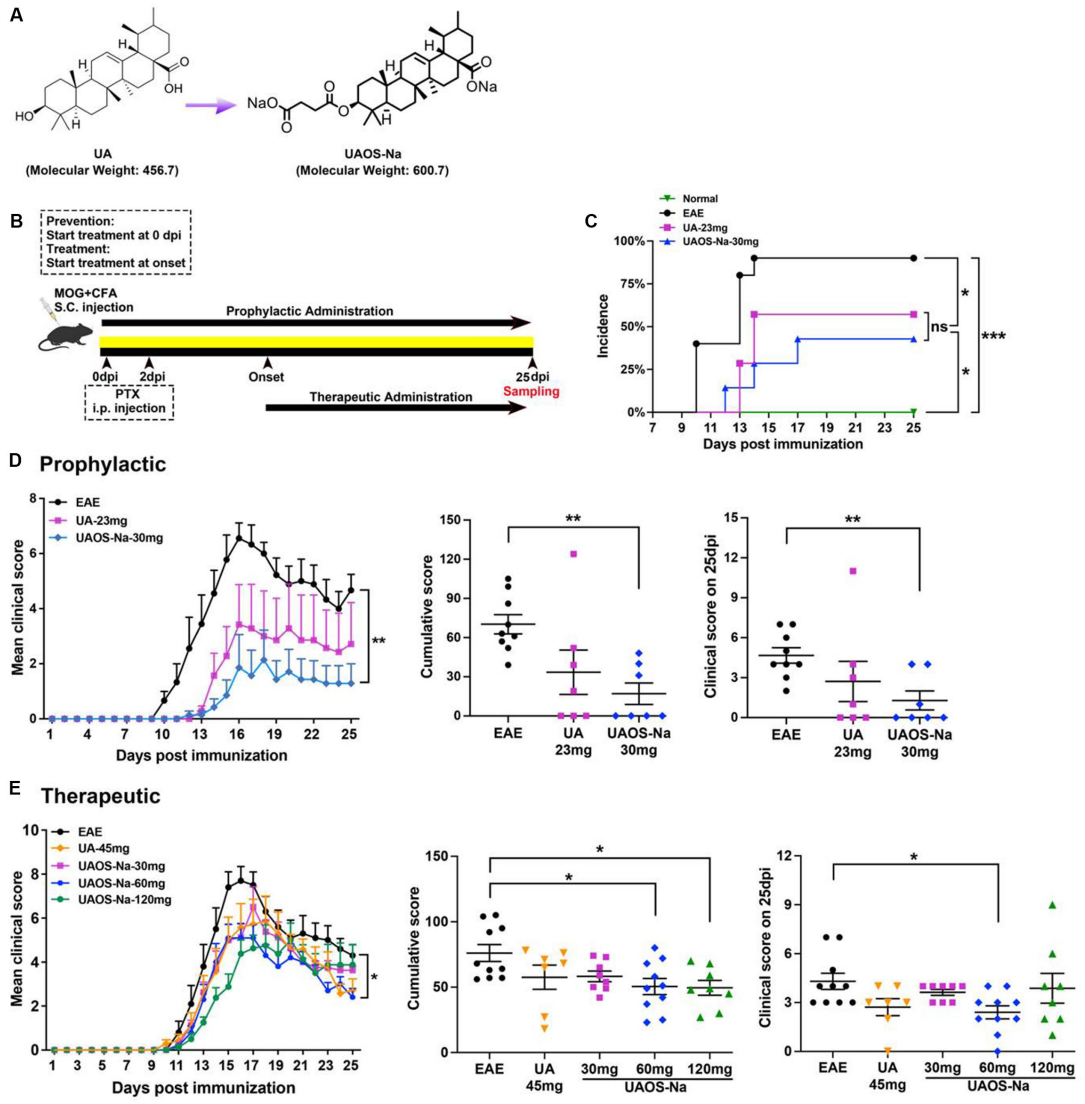
Preliminary comparison of pharmacodynamics between UA and UAOS-Na in EAE. **(A)** Chemical structures of UA (molecular weight: 456.7) and UAOS-Na (molecular weight: 600.75). Female, 6 to 8 weeks-old C57BL/6J mice were immunized with MOG35-55 and treated with UA and UAOS-Na by oral gavage. **(B)** UA and UAOS-Na were used for EAE prophylactic/therapeutic; *n* = 7–10 in each group. Incidence **(C)** and mean clinical score **(D)** of EAE mice administrated prophylactically with UA (23 mg/kg/d) and UAOS-Na (30 mg/kg/d). In **(C)**, incidence of EAE mice, log-rank (Mantel–Cox) test, and simple survival analysis (Kaplan–Meier) were performed, ^*^p < 0.05. **(E)** Mean clinical score of EAE mice treated with UA (45 mg/kg/d) and UAOS-Na at different doses (30, 60, 120 mg/kg/d). Data were expressed as mean ± SEM. Compared with EAE group, one-way ANOVA with Tukey’s multiple comparisons test, ^*^p < 0.05 and ^**^p < 0.01.

UAOS-Na, batch number: 20200706, prepared by the Shanghai University of Traditional Chinese Medicine, and the voucher specimen (No. UAOS-Na20200706) was deposited at the Department of TCM Chemistry, Pharmacy of Shanghai University of Traditional Chinese Medicine (Shanghai, China). In our previous work, UAOS-Na was synthesized by forming a C-3 hydroxyl ester of UA with succinic acid and then alkalizing to a salt, and its structure was confirmed by ^1^H-NMR, ^13^C-NMR and LCMS (ESI) (Figure 1A). The water solubility of UAOS-Na (577.58 mg/L) was determined by using ultraviolet-visible spectrophotometry and was significantly higher than that of UA [<5.64 mg/L (29)]. In this study, UAOS-Na was dissolved in 0.5% sodium carboxymethyl cellulose (CMC-Na, #abs47051270; Absin Bioscience, Shanghai, China) in the comparative efficacy experiments with UA (#SLURA190502; Sanleng Biotech, Guilin, China). Otherwise, UAOS-Na was dissolved in ddH_2_O.

### UA/UAOS-Na prophylactic/therapeutic administration

For prophylactic administration experiments (Figure 1B), mice were randomly divided into a normal group (*n* = 6, 0.5% CMC-Na), an EAE group (*n* = 10, EAE induction, 0.5% CMC-Na), a UA group (*n* = 7, EAE induction, 23 mg/kg/d), and a UAOS-Na group (*n* = 7, EAE induction, 30 mg/kg/d, the molar concentration of UAOS-Na was consistent with UA). UA or UAOS-Na was prepared with 0.5% CMC-Na. Mice were treated with 0.5% CMC-Na, UA, or UAOS-Na by gavage daily from the beginning of modeling (0 dpi). Asymptomatic animals were included in the statistical analysis.

For therapeutic administration experiments (Figure 1B), mice were randomly divided into a normal group (*n* = 11, 0.5% CMC-Na), an EAE group (*n* = 10, EAE induction, 0.5% CMC-Na), a UA group (*n* = 7, EAE induction, 45 mg/kg/d), and UAOS-Na groups [EAE induction, 30 mg/kg/d (*n* = 9), 60 mg/kg/d (*n* = 10), 120 mg/kg/d (*n* = 8)]. UA or UAOS-Na was prepared with 0.5% CMC-Na. Mice were given individualized treatment, that is, mice were treated with 0.5% CMC-Na, UA, or UAOS-Na only after they developed clinical symptoms (onset, 10–14 dpi). Mice that did not onset until 15 dpi after modeling were considered asymptomatic animals and were not included in the statistical analysis.

Mice were anesthetized and sacrificed at 25 dpi and perfused with 4% paraformaldehyde (PFA; #G1101; Servicebio, Wuhan, China). Their serum, spinal cord, and spleen were harvested. The spleens were isolated to calculate the spleen index (SI) as follow: SI = spleen weight (mg)/body weight (g) × 10.

### Cuprizone-induction and UAOS-Na treatment

After 1 week of adaptive feeding, male C57BL/6 mice were randomly divided into a normal group (10 kcal% Fat Diets, Research Diets, United States) given ddH_2_O, a cuprizone-induction group [CPZ group, 0.2% cuprizone (w/w), Research Diets] given ddH_2_O, a UAOS-Na group [0.2% cuprizone (w/w), Research Diets] given UAOS-Na (60 mg/kg/d). These mice in CPZ and UAOS-Na groups were fed cuprizone for 6 weeks to achieve complete demyelination of corpus callosum. From the 2nd week, ddH_2_O or UAOS-Na (60 mg/kg/d) was administered daily by gavage. At the 6th week, the mice were sacrificed, and brain were obtained.

### Preparation of spleen lymphocyte

Spleens were mechanically dissociated through a 70 μm cell strainer (#02036461; Titian, Shanghai, China) and incubated with red blood cell lysis buffer (#C3702; Beyotime, Shanghai, China) for 5 min. Harvested cells were washed with cold PBS and fractionated on lymphocyte separation reagent (#LSM01; Multisciences Biotech, Zhejiang, China) gradient by centrifugation at 400 × *g* for 30 min. Lymphocyte were obtained by collecting mononuclear cells (MNCs) from the middle layer and washing with PBS.

### Induction of murine bone marrow derived dendritic cells

Bone marrow cells (BMC) were extracted from the femurs and tibiae of C57BL/6 mice under sterile conditions, lysed with red blood cell lysis buffer for 10 min, centrifuged at 400 × *g* at 4°C for 5 min, and washed twice with RPMI 1640 medium (#MA0215; Meilunbio, Dalian, China). Cells were resuspended in DCs complete culture medium consisting of granulocyte-macrophage colony-stimulating factor (#70343; CST, Massachusetts, United States, 20 ng/mL), interleukin-4 (#abs04697, Absin, 10 ng/mL), and RPMI 1640 complete medium, and adjusted to 1 × 10^6^ cells/mL. Cells were seeded into 6-well plate and cultured for 72 h. Adherent cells were retained, supplemented with fresh DCs complete medium, and cultured for 48 h. Half of the medium was replaced with DCs complete medium for an additional 48 h. On day 7, suspension cells, immature bone marrow-derived dendritic cells (imBMDC), were collected and reseeded in 6-well plate and stimulated with LPS (100 ng/mL) for 24 h to obtain mature mouse bone marrow-derived dendritic cells (mBMDC). In the UAOS-Na groups, different concentrations of UAOS-Na (0, 10, 20, and 40 μM) were added to the culture medium together with LPS (100 ng/mL) for 24 h.

### MOG-mBMDC was co-cultured with MOG-lymphocyte

MOG-specific mBMDC (MOG-mBMDC) was obtained by stimulating imBMDC with MOG35-55 (20 μg/mL) for 4 h followed by LPS (100 ng/mL) for 24 h and centrifuged at 400 × *g* at 4°C for 5 min. MOG-specific lymphocytes (MOG-lymphocytes) were obtained from EAE mouse spleen according to the splenic lymphocyte preparation protocol. MOG-mBMDC and MOG-lymphocytes were co-cultured at a concentration of 1:10 in DCs complete culture medium containing IL-2 (#abs04100; Absin, 10 ng/mL) for 48 h. Then, cell counting kit-8 (CCK-8) and flow cytometry were performed.

In the UAOS-Na groups, different concentrations of UAOS-Na (0, 10, 20, and 40 μM) were added to the culture medium together with LPS (100 ng/mL) for 24 h.

### Cytokine measurement by enzyme-linked immunosorbent assay

After anesthesia, mouse blood was collected by removing eyeballs. Serum was separated by centrifugation at 1,000 × *g* for 10 min. To evaluate the peripheral inflammation in mice, serum levels of interferon-γ (IFN-γ; #EK280; Multisciences Biotech), tumor necrosis factor α (TNF-α; #EK282; Multisciences Biotech), interleukin-17A (IL-17A, #EK217; Multisciences Biotech), interleukin-6 (IL-6, #EK206; Multisciences Biotech), and interleukin-10 (IL-10, #EK210; Multisciences Biotech) were determined using commercial enzyme-linked immunosorbent assay (ELISA) kits.

### Histopathological and immunohistochemical analysis

The spinal cords were harvested, fixed with 4% PFA, embedded in paraffin, and sliced into 4 μm-thick sections for pathological analysis. To determine the degree of inflammatory infiltration (MNCs infiltration), spinal cord sections were stained with hematoxylin and eosin (H&E, #G1005; Servicebio). Images were obtained with a 10× objective of Lecia microscope at a 100× magnification. The white matter in transverse spinal cord tissue sections was divided into the following parts, dorsal columns, spinocerebellar, spinothalamic, ventral corticospinal tract, and vestibulospinal, all parts were assessed and scored in a blind manner. Finally, the scores of each part were averaged. The scoring criteria for tissue inflammation were as follow: 0 = none, 1 = some scattered MNCs, 2 = MNCs infiltrating around blood vessels and forming “cuff-like changes” of blood vessel, 3 = numerous “cuff-like changes” were formed and MNCs infiltrated to adjacent tissues (24, 30). To determine the degree of demyelination, spinal cord sections were stained with luxol fast blue (LFB, #G1030; Servicebio). For semiquantitative methods of demyelination (LFB staining), images were obtained with a 10× objective of Lecia microscope at a 100× magnification, the spinal cord transection was divided into four parts, front, back, left, and right. These four visions were selected to calculate degree of LFB staining (myelination area) in each group and the average was taken as the final count. The degree of LFB staining was analyzed automatically using Image-Pro Plus 6.0 software.

The brains of CPZ mouse were harvested, fixed with 4% PFA, embedded in paraffin, and sliced into 4 μm-thick sections for pathological analysis. To determine the degree of corpus callosum demyelination, brain sections were stained with LFB, images were then obtained using a Lecia microscope and quantified using Image-Pro Plus 6.0 software.

Spinal cord of EAE or brain sections of CPZ mice were immunostaining with glial fibrillary acidic protein (GFAP, #80788; CST; 1:200) to evaluate astrocytic lesions. Images were then obtained using a Lecia microscope and quantified using Image-Pro Plus 6.0 software.

### Immunofluorescence

Transverse sections of spinal cord were cut. After antigen repair and endogenous peroxidase removal, slices were blocked in 3% bovine serum albumin (BSA, #MB4219; Meilunbio) for 1 h. Primary Abs, major histocompatibility complex class I (MHC-I, #NBP3-09017; NOVUS, Beijing, China; 1: 300) and CD8a (#GB114196; Servicebio; 1: 5000), incubations were conducted overnight at 4°C. HRP labeled secondary Abs incubations were conducted 50 min at room temperature, followed by washing with PBS 3 times. To amplify signal, slices were stained with TSA (#G1223; Servicebio) for 10 min at room temperature. After washing with PBS 3 times, nuclei were stained with DAPI (#G1012; Servicebio). Slides were covered with anti-fluorescence quenching tablet seal (#G1401; Servicebio). Images were then obtained using multispectral tissue slice scanning system and quantified using Image-Pro Plus 6.0 software.

### Transmission electron microscope

Mice were deeply anesthetized and perfused with 4% PFA, 1.5% glutaraldehyde and 1 mM CaCl_2_ in 0.1 M cacodylate buffer. Spinal cord sections were harvested and fixed in the same solution at 4°C for 24 h. Sections were washed, post-fixed with 1% OsO_4_ in 0.1 M PBS (pH 7.4) for 2 h at room temperature, and subsequently dehydrated in graded ethanol series. Embedding was performed in epoxypropane and embedding agent. Sections, 1.0 μm thick, were cut, stained in toluidine blue (1%), and examined by light microscopy (CKX41, Olympus, Japan) to locate the spinothalamic tract. Ultrathin sections (60 nm) were cut, dyed in 2% uranium acetate saturated for 8 min in dark, and in 2.6% lead citrate solution for 8 min without CO_2_. Then, sections were viewed and photographed with a transmission electron microscope (FEI Tecnai G2 spirit, Leica, Germany) operated at 80 kV. For each sample at 8200× magnification, four fields (up, down, left, and right) in the spinothalamic tract were obtained for statistical analysis. Quantification of the thickness (G-ratio = axon diameter/fiber diameter) and the number of myelinated fibers in the spinothalamic plexus were analyzed by Image-Pro Plus 6.0 software.

### Real-time PCR

Total RNA was extracted from spinal cords or spleens using RNA isolater Total RNA Extraction Reagent (#R401-01, Vazyme, Nanjing, China) according to the manufacturer’s instructions. Reverse transcription was conducted using 5 × HiScript II qRT SuperMix II (#R223-01; Vazyme). Real-Time PCR was performed using the ChamQ Universal SYBR qPCR Master Mix (#Q711; Vazyme) according to the manufacturer’s instructions, and detection was performed using the ABI Prism^®^ 7500 Sequence Detection System (Applied Biosystems, Foster City, CA, United States). All data were normalized to an average of a housekeeping genes *Gapdh*. Gene relative expression was calculated by log2 of −ΔΔCt values from triplicate of PCR.

**Table 1:** 

Genes	Primers (5′-3′)
M-GAPDH-F	TCTCCTGCGACTTCAACA
M-GAPDH-R	TGTAGCCGTATTCATTGTCA
M-H2-T23-F	GCCTCACAGATCTCTAAGCACA
M-H2-T23-R	CAGCACCTCAGGGTGACTTC
M-Rorc-F	CCTACTGAGGAGGACAGGGA
M-Rorc-R	GCAGGAGTAGGCCACATTACA
M-Tapbp-F	GTCTCCCTCGCGGACTAAAG
M-Tapbp-R	CCACACGGGGACATTCGTAA
M-T-bet-F	CCTTCTCACCTCTTCTATCC
M-T-bet-R	AACTCCGCTTCATAACTGT

### Western blot analysis

The spinal cord was collected in accordance with standard procedures. Protein lysates were then obtained in RIPA lysis buffer (#P0013B; Beyotime) containing protease and phosphatase inhibitors. The concentration of these protein lysates was then measured using a Bicinchoninic Acid Protein Assay Kit (#P0012S; Beyotime). Equal amounts (3 μg/μL, 10 μL) of proteins in each sample were loaded onto 10% SDS-PAGE gels (#P0690; Beyotime) and transferred to polyvinylidene fluoride membranes (Millipore, Burlington, MA, United States). The membranes were then blocked with 5% BSA and probed with different primary antibodies: MHC-I (#ab281904, Abcam, Waltham, MA, United States), Tapbp (#66382; CST), GAPDH (#abs132004; Absin), Tubulin (#2148; CST), and anti-rabbit secondary antibody (#ab205718; Abcam). Blots were then exposed to an enhanced chemiluminescence detection reagent (#P0018S; Beyotime). Finally, signals were visualized using a Tanon imaging system and quantified using Image-Pro Plus 6.0 software.

### Flow cytometry

For surface-marker staining, cells were incubated with fluorochrome-conjugated Abs to CD4 (#100405; Biolegend, California, United States), CD8a (#162304; Biolegend), CD3ε (#100312; Biolegend), CD11c (#117309, Biolegend), CD86 (#159203; Biolegend), and MHC-I (#114606; Biolegend) at the recommended dilution 30 min on ice. Flow cytometry analysis was performed on Beckman CytoFlex S (Beckman, California, United States) and data were analyzed with FlowJo software (Treestar, Ashland, Kentucky, United States).

### Tandem mass tag quantitative proteomics

#### Protein extraction, digestion, and tandem mass tag labeling

Nine spinal cord tissue samples (4 mice spinal cords were mixed into one sample, 3 samples per group) collected from the normal group, EAE group, and UAOS-Na group (60 mg/kg/d) were mixed with SDT buffer and homogenized using SCIENTZ-48 High Throughput Tissue Grinder (SB-3200DT, Xinzhi, Zhejiang, China). The homogenate was cracked on ice for 30 min and centrifuged at 12000 × *g* for 20 min. The supernatant was collected, and the protein concentration was detected by a BCA kit (#P0012S; Beyotime). For digestion, the protein solution which containing 100 μg protein was reduced with 2 μL 0.5 M TCEP (#646547; Sigma) at 37°C for 60 min and alkylated with 4 μL 1 M iodoacetamide (#I1149; Sigma) at room temperature for 40 min in darkness. Fivefold volumes of cold acetone were added to precipitate protein at −20°C overnight. After centrifugation at 12000 × *g* at 4°C for 20 min, the samples were washed twice by 1 mL pre-chilled 90% acetone aqueous solution. Trypsin (#G3440; Servicebio) was added at 1:50 trypsin-to-protein mass ratio and incubated at 37°C overnight. The peptide mixture was desalted by C18 ZipTip and quantified by Pierce^™^ Quantitative Colorimetric Peptide Assay (#23275; Thermo Fisher, Massachusetts, United States) and then lyophilized. Trypsin-digested peptides were labeled with TMT-10 Plex (#90111; Thermo Fisher) reagents according to the manufacturer’s instructions.

#### High pH reversed-phase fractionation and LC-MS analysis

The peptide mixture was re-dissolved in the buffer A (20 mM ammonium formats in water, pH 10.0, adjusted with ammonium hydroxide), and then fractionated by high pH separation using Ultimate 3000 system (Thermo Fisher) connected to a reverse phase column (XBridge C18 column, 4.6 mm × 250 mm, 5 μm), (Waters Corporation, MA, United States). High pH separation was performed using a linear gradient, starting from 5% B to 45% B in 40 min (B: 20 mM ammonium formate in 80% ACN, pH 10, adjusted with ammonium hydroxide). The column was re-equilibrated at the initial condition for 15 min. The column flow rate was maintained at 1 mL/min and the column temperature was maintained at 30°C. Ten fractions were collected; each fraction was dried in a vacuum concentrator for the next step. The peptides were re-dissolved in solvent A (0.1% formic acid in water) and analyzed by Orbitrap Fusion™ Lumos^™^ Tribrid^™^ coupled to an EASY-nanoLC 1,200 system (Thermo Fisher). Two microliter peptide sample was loaded onto a 25 cm analytical column [75 μm inner diameter, 1.9 μm resin (Dr. Maisch, Tübingen, Germany)] and separated with 120 min-gradient starting at 4% buffer B (80% ACN with 0.1% FA) for 4 min followed by a stepwise increase to 30% in 104 min, 90% in 2 min and stayed there for 10 min. The column flow rate was maintained at 600 nL/min with the column temperature of 55°C. The electrospray voltage was set to 2 kV. The mass spectrometer was run under data dependent acquisition (DDA) mode, and automatically switched between MS and MS/MS mode. The survey of full scan MS spectra (*m*/*z* 350–1,500) was acquired in the Orbitrap with 60,000 resolutions. The automatic gain control (AGC) target of 4e5 and the maximum injection time of 50 ms. Then the precursor ions were selected into collision cell for fragmentation by higher-energy collision dissociation (HCD), the normalized collection energy was 40. The MS/MS resolution was set at 50,000, the automatic gain control (AGC) target of 5e4, the maximum injection time of 50 ms, and dynamic exclusion was 30 s.

#### Database searching

Tandem mass spectra were processed by PEAKS Studio version 10.6 (Bioinformatics Solutions Inc., Waterloo, Canada). PEAKS DB was set up to search the database of uniport-*Mus musculus* (version 201907, 22290 entries) assuming trypsin as the digestion enzyme. PEAKS DB were searched with a fragment ion mass tolerance of 0.02 Da and a parent ion tolerance of 10 ppm. Carbamidomethylation (C) and TMT-10 plex were specified as the fixed modification. Oxidation (M) and Acetylation (Protein N-term) were specified as the variable modifications. Peptides were filtered by 1% FDR and proteins were filtered by 1 unique peptide. Reporter ions were used to calculate the quantification ratio between samples. Normalization was calculated from the total intensity of all labels in all quantifiable peptides. After *t*-test, different expressed proteins were selected if their *p* < 0.05 and |fold change| >1.5.

#### Bioinformatics analysis

Differential expression proteins (DEPs) were subjected to multiple bioinformatic analyses. Gene Ontology (GO) annotation and Kyoto Encyclopedia of Genes and Genomes (KEGG) pathways enrichment analysis was performed using Database for Annotation, Visualization, and Integrated Discovery Bioinformatics Resources.^1^

### Statistical analysis

Statistical analyses were performed using GraphPad Prism 8 software (GraphPad, La Jolla, CA, United States). The results are presented as mean ± SEM. Group comparisons were established by one-way analysis of variance (ANOVA) with Tukey’s multiple comparisons test. Student’s *t*-test was used for small sample sizes, and the normality of data was analyzed using the Kolmogorov–Smirnov test. A *p*-values less than 0.05 was considered statistically significant.

## Results

### Preliminary comparison of pharmacodynamics between UA and UAOS-Na in EAE

For prophylactic administration, EAE mice were given UA (23 mg/kg/d) and UAOS-Na (30 mg/kg/d) for prophylactic until 25 dpi (Figure 1B). In EAE group, mice began to develop the disease (onset) at 10 dpi, with an average onset time of 11.78 ± 0.57 dpi and an incidence of 90.0% (9/10). UA and UAOS-Na prophylactic administration significantly delayed the average onset time to 13.50 ± 0.29 dpi and 14.33 ± 1.45 dpi and reduced the incidence to 57.1% (4/7) and 42.9% (3/7), respectively (Figure 1C). In addition, UAOS-Na significantly reduced the cumulative score and 25 dpi score and improved the symptoms of EAE mice comparing with normal group, but UA just had a weakening trend in the same experiment (Figure 1D). UAOS-Na may be more effective than UA in preventing EAE.

**Figure 2:**
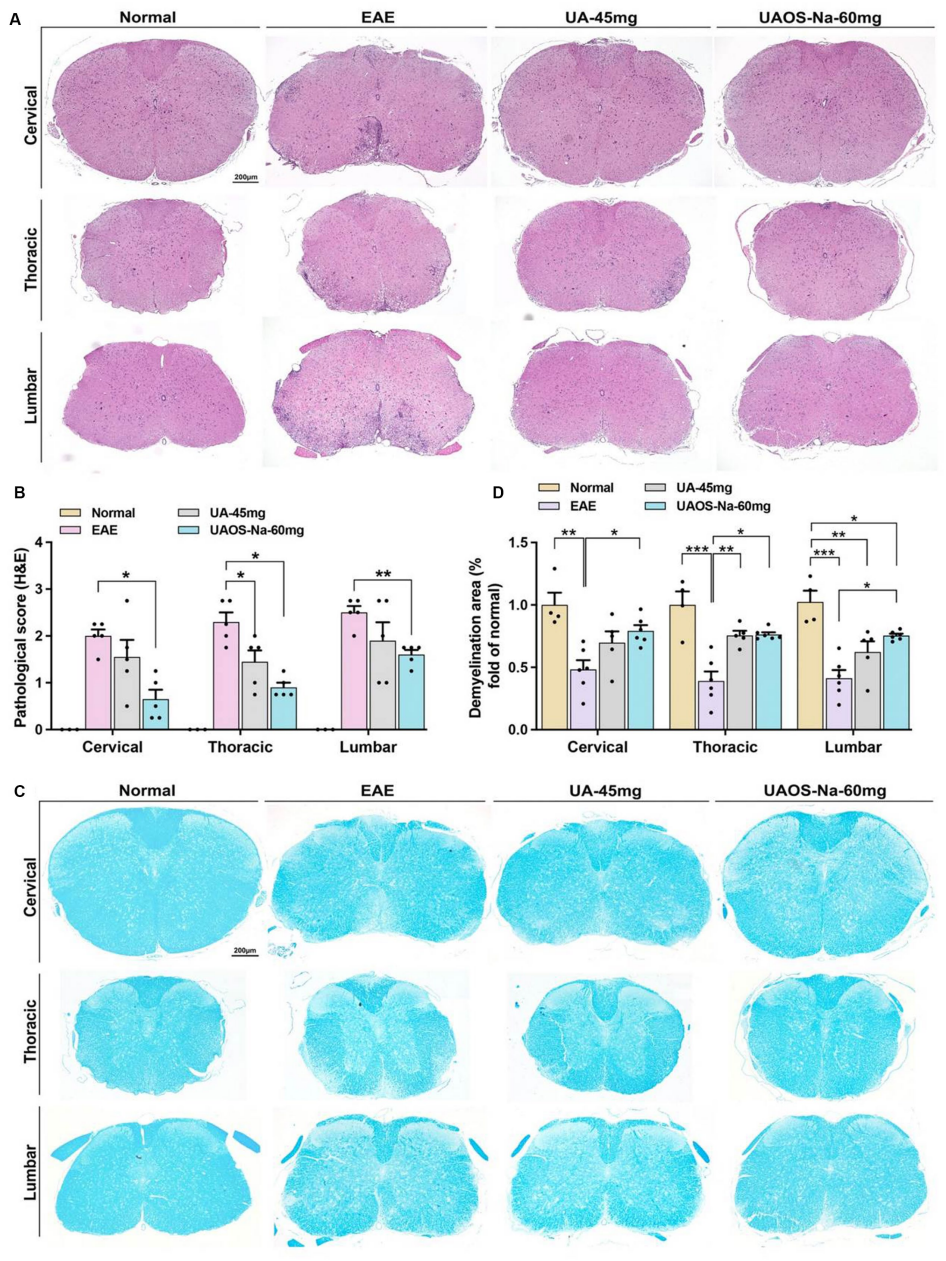
UAOS-Na reduces CNS inflammation and demyelination of EAE mice. Female, 6 to 8 weeks-old C57BL/6J mice were immunized with MOG35-55 and treated with UAOS-Na (60 mg/kg/d) and UA (45 mg/kg/d) from onset to 25 dpi. **(A)** Sections (cervical, thoracic, and lumbar spinal cords) were assayed for inflammation by H&E (scale bar = 200 μm), and **(B)** CNS pathology was scored. **(C)** Sections (cervical, thoracic, and lumbar spinal cord) were assayed for demyelination by LFB (scale bar = 200 μm), and **(D)** CNS pathology was analyzed automatically using Image-Pro Plus 6.0 software. Data were expressed as mean ± SEM; *n* = 3–6 in each group, one-way ANOVA with Tukey’s multiple comparisons test was performed. ^*^p < 0.05, ^**^p < 0.01, and ^***^p < 0.001.

For therapeutic administration, UA (45 mg/kg/d) and different doses of UAOS-Na (30, 60, 120 mg/kg/d) were administered to EAE mice. UAOS-Na 60 mg/kg/d significantly reduced both the cumulative score and the 25-dpi score of EAE mice. Just like the result of prophylactic administration in Figure 1D, there was only significantly change between UAOS-Na-60 mg/kg/d group and normal group (Figure 1E). Moreover, the effects of UAOS-Na (60 mg/kg/d) and UA (45 mg/kg/d) on inflammation (H&E) and demyelination (LFB) of EAE spinal cords were compared. The results showed that UAOS-Na was more effective than UA in alleviating spinal cord inflammation and demyelination in EAE mice (Figure 2), and UAOS-Na 60 mg/kg/d was used in subsequent *in vivo* experiments.

### UAOS-Na ameliorates peripheral and CNS inflammation in EAE mice

**Figure 3:**
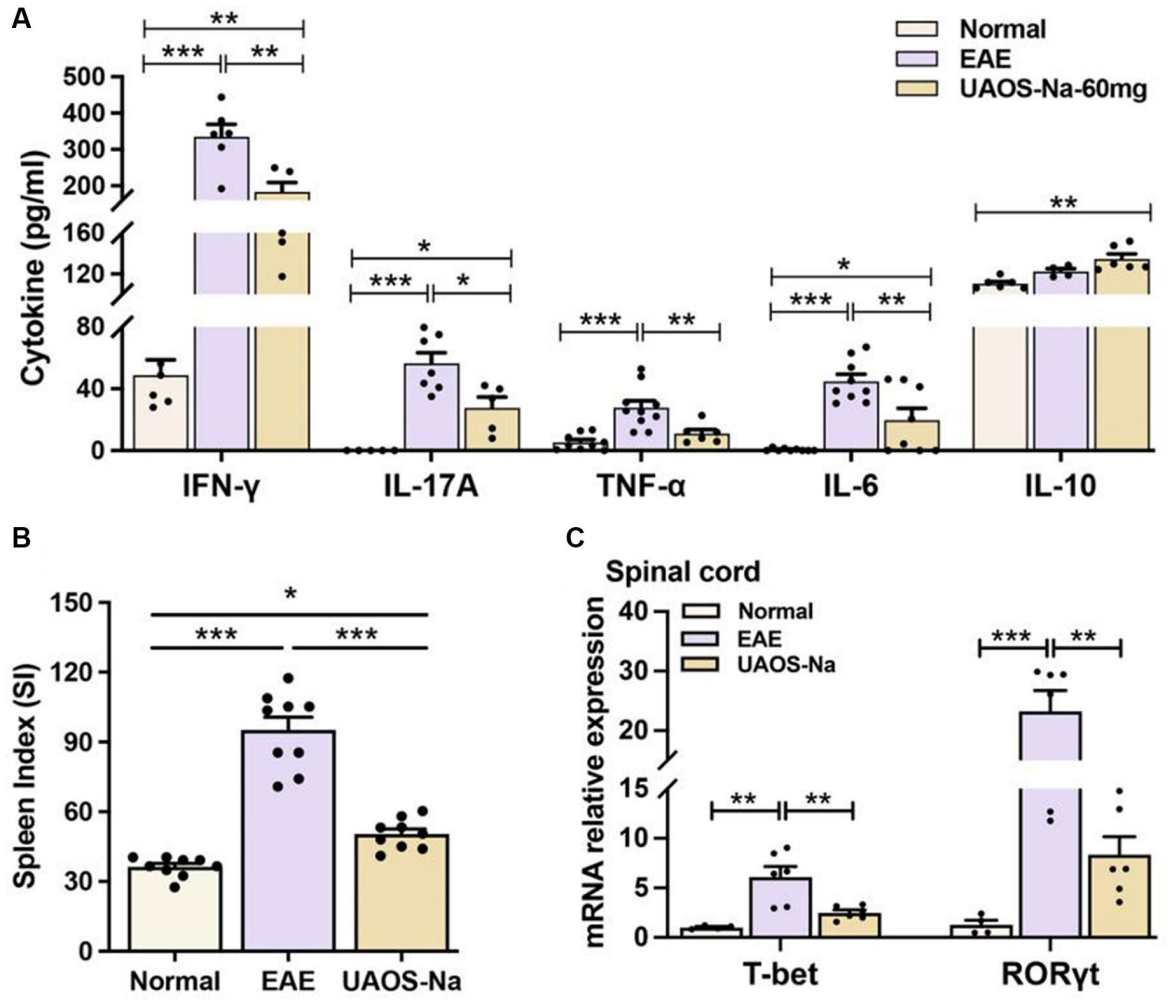
UAOS-Na ameliorates inflammation in EAE mice. Female, 6 to 8 weeks-old C57BL/6J mice were immunized with MOG35-55 and treated with UAOS-Na 60 mg/kg/d from onset to 25 dpi. **(A)** Serum levels of cytokines IFN-γ, IL-17A, TNF-α, IL-6 and IL-10 analyzed by ELISA, *n* = 5–10. **(B)** Spleen index of mice, *n* = 9. **(C)** mRNA levels of T-bet and ROR-γt of mouse spinal cord analyzed by real-time PCR, *n* = 4–6. Data were expressed by mean ± SME; one-way ANOVA with Tukey’s multiple comparisons test was performed. ^*^p < 0.05, ^**^p < 0.01, and ^***^p < 0.001.

The imbalance of serum pro-inflammatory and anti-inflammatory cytokines is closely related to EAE inflammation. Literatures have reported that inhibiting the expression of pro-inflammatory cytokines such as IFN-γ, TNF-α, IL-6, and IL-17 (31, 32) and promoting the expression of anti-inflammatory cytokines such as IL-10 (33, 34) can alleviate the inflammation of EAE. Moreover, small molecules targeting T-bet and RORγt also inhibit EAE (35). Therefore, the serum levels of cytokines were measured by ELISA and the spleen index of mice was recorded. The results showed that UAOS-Na (60 mg/kg/d) significantly reduced serum levels of the pro-inflammatory cytokines IFN-γ, IL-17A, TNF-α and IL-6, increased the serum level of anti-inflammatory cytokine IL-10 (Figure 3A), and reduced the spleen index (Figure 3B). In parallel, real-time PCR was used to detect the mRNA levels of *T-bet* and *ROR-γt*. UAOS-Na obviously reduced the mRNA levels of *T-bet* and *ROR-γt* of EAE spinal cord tissues (Figure 3C). Combined with the results in Figures 2A,B, UAOS-Na reduced peripheral and CNS inflammation in the acute stage of EAE mice.

### UAOS-Na reduces demyelination in EAE mice and protects the myelin sheath in cuprizone-induced mice

**Figure 4:**
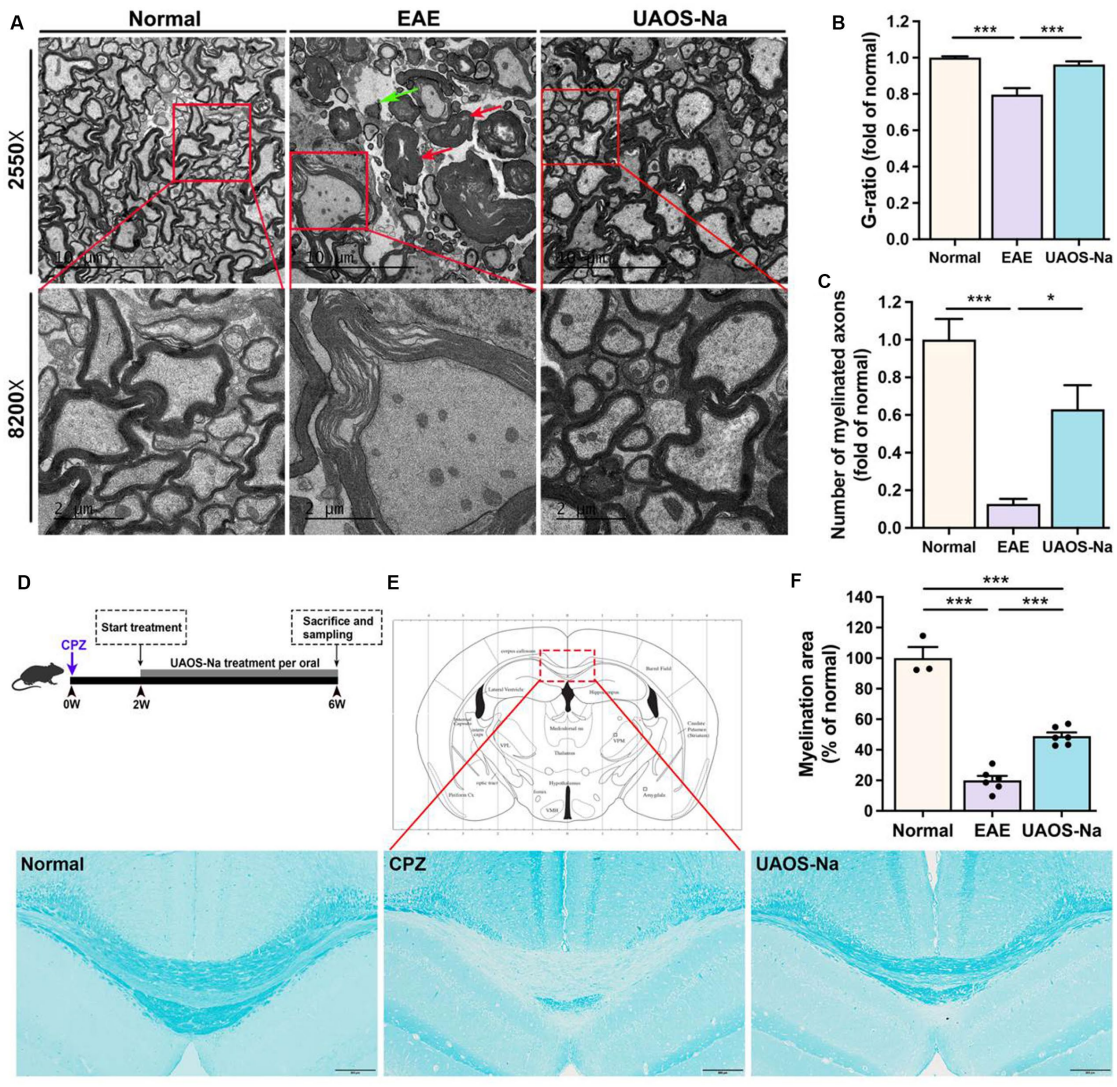
UAOS-Na reduces demyelination in EAE mice and protects the myelin sheath in Cuprizone-induced mice. **(A–C)** Female, 6 to 8 weeks-old C57BL/6J mice were immunized with MOG35–55 and treated with UAOS-Na (60 mg/kg/d) from onset to 25 dpi, *n* = 5–6 in each group. **(A)** Ultrastructure of the spinothalamic nerve cluster in the transverse section of the lumbar spinal cord measured by transmission electron microscope. The red arrow showed myelin edema; the green arrow showed axon demyelination (scale bar = 10 μm in the upper images and 2 μm in the lower images in **A**). Quantification of the G-ratio (axon diameter/fiber diameter, **B**) and the number **(C)** of myelinated fibers in the spinothalamic plexus. **(D)** Male, 6 to 8 weeks-old C57BL/6J mice were fed with cuprizone for 6 weeks to obtain complete demyelination in the corpus callosum, *n* = 3–5 in each group; UAOS-Na was given daily from the 2nd week. **(E)** The corpus callosum was assayed for demyelination by LFB, and **(F)** CNS pathology was analyzed automatically using Image-Pro Plus 6.0 software (scale bar = 200 μm in E). Data were measured by mean ± SME; one-way ANOVA with Tukey’s multiple comparisons test was performed. ^*^p < 0.05 and ^***^p < 0.001.

To evaluate the effects of UAOS-Na on myelin sheath at the acute stage of EAE mice, LFB staining and transmission electron microscope for EAE spinal cord were performed. As shown in Figures 2C,D, the LFB staining in EAE group showed obvious demyelinating lesions in the cervical, thoracic, and lumbar spinal cords. In UAOS-Na group, demyelinating lesions were significantly reduced. Moreover, spinothalamic nerve cluster ultrastructure in the transverse section of lumbar spinal cord was measured by transmission electron microscope. As shown in Figures 4A–C, compared with normal group, the EAE group showed discrete stratification and edema of axon’s myelin sheath, as well as a reduction in the number of myelinated axons. The above data supported that UAOS-Na reduced axonal myelin injury in the acute stage of EAE mice.

UAOS-Na alleviated demyelination in EAE mice, which might be secondary to its immunomodulatory effect. To test whether UAOS-Na has a direct effect on myelin, we used the cuprizone-induced demyelination mouse model with minimal inflammation (Figure 4D). At 6th week after modeling, mouse brains were harvested for LFB staining to analyze the degree of demyelination in the corpus callosum. The results showed that CPZ group showed obvious demyelination compared with normal group. However, compared with CPZ group, demyelination was significantly alleviated in UAOS-Na group (Figures 4E,F). Therefore, UAOS-Na could directly protect the myelin sheath in cuprizone-induced mice.

### UAOS-Na ameliorates astrogliosis in EAE mice and cuprizone-induced mice

**Figure 5:**
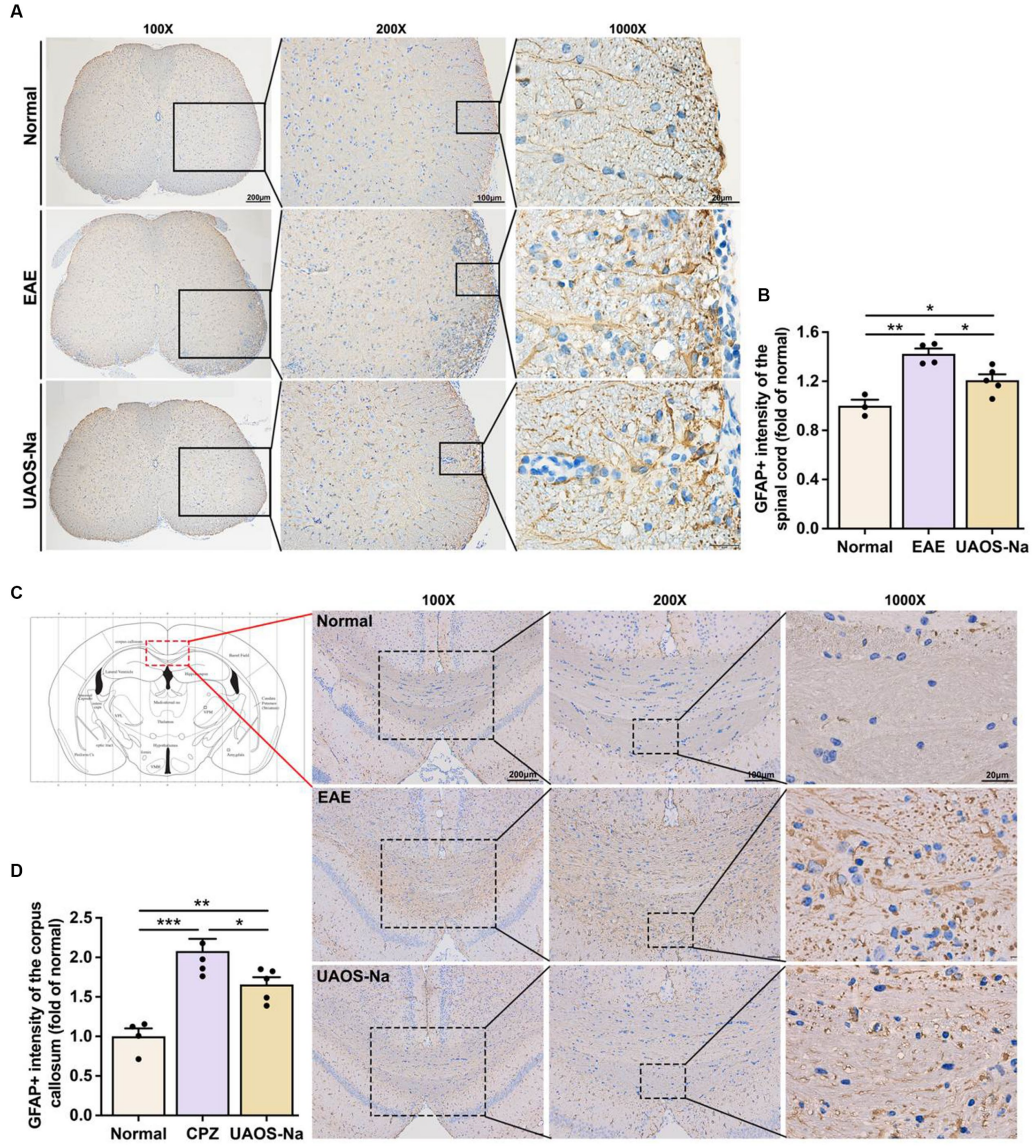
UAOS-Na rameliorates astrogliosis in EAE and cuprizone-induced mice. **(A,B)** Female, 6 to 8 weeks-old C57BL/6J mice were immunized with MOG35-55 and treated with UAOS-Na (60 mg/kg/d) from onset to 25 dpi. Immunohistochemistry of the spinothalamic nerve cluster in the transverse section of lumbar spinal cord (**A**, scale bar = 200 μm on the left, 100 μm in the middle, and 20 μm on the right) and the quantitative analysis of GFAP expression using Image-Pro **(B)**, *n* = 3–5 in each group. **(C,D)** Male, 6 to 8 weeks-old C57BL/6J mice were fed with cuprizone for 6 weeks to obtain complete demyelination in the corpus callosum; UAOS-Na was given daily from the 2nd week. Immunohistochemistry of the corpus callosum (**C**, scale bar = 200 μm on the left, 100 μm in the middle, and 20 μm on the right) and the quantitative analysis of GFAP expression using Image-Pro **(D)**, *n* = 5–6 in each group. Data were measured by mean ± SME; one-way ANOVA with Tukey’s multiple comparisons test was performed, ^*^p < 0.05, ^**^p < 0.01, and ^***^p < 0.001.

The lesions of AST may be an important reason for the failure of remyelination. Studies have shown that reactive AST forms the physical barrier and secretes soluble factors to inhibit remyelination (36–38). So, we determined the effects of UAOS-Na on CNS astrogliosis in EAE mice and CPZ mice. Immunohistochemical staining of the lumbar spinal cord of EAE mice and the corpus callosum of CPZ mice showed deepened GFAP staining, massive reactive astrogliosis, and glial scar formation, which were reversed by UAOS-Na treatment (Figures 5A,C). And the data measured by Image-Pro showed that UAOS-Na treatment significantly reduced reactive astrogliosis and glial scar formation of EAE and CPZ mice (Figures 5B,D). Therefore, UAOS-Na ameliorated astrogliosis in the acute stage of EAE mice and prevented astrogliosis in CPZ mice.

### TMT-based proteomic analysis of the mouse spinal cord

**Figure 6:**
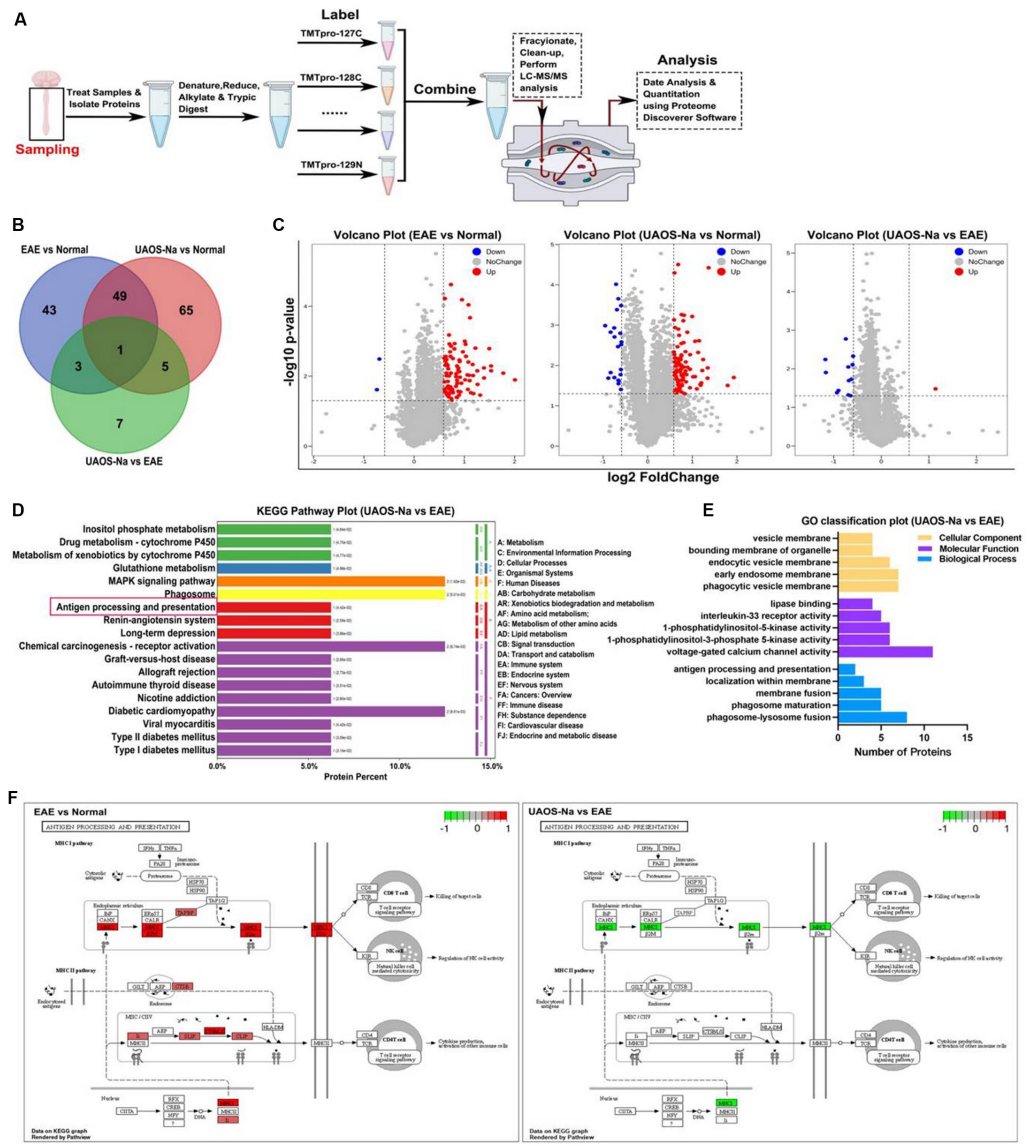
TMT-based proteomic analysis of mouse spinal cords. Female, 6 to 8 weeks-old C57BL/6 J mice were immunized with MOG35-55 and treated with UAOS-Na (60 mg/kg/d) by oral gavage from onset to 25 dpi. **(A)** Overview of TMT-based spinal cord analysis. Venn map **(B)** and volcano map **(C)** of DEPs in three comparison groups. In **(C)**, log2 of FC as the horizontal axis and -log10 of *p*-value as the vertical axis, red dot means up-regulation, blue dot means down-regulation. **(D)** Histogram of KEGG enrichment of DEPs in UAOS-Na vs. EAE group (Top 20). The ordinate on the left shows the signaling pathways. The ordinate on the right, from outside to inside, represents the abbreviation of the primary and secondary classification names of the signaling pathways. The number in brackets shows the *p*-value. **(E)** GO functional annotation of DEPs in UAOS-Na vs. EAE group (associated with antigen processing and presentation). **(F)** DEPs in the APP signaling pathway in EAE vs. Normal and UAOS-Na vs. EAE groups. The red box indicates up-regulated DEPs, while the green box indicates down-regulated DEPs.

Proteomic technique based on mass spectrometry has been widely used in searching for therapeutic targets of diseases (39, 40). In this study, tandem mass tag (TMT) technique was performed for proteomic analysis of the mouse spinal cord tissues (Figure 6A), and the data were presented in Supplementary Tables S1–S3. DEPs were defined as *p* < 0.05 and |fold change (FC)| >1.5. Compared with normal mice, 96 DEPs were enriched in EAE mice, of which 94 DEPs were upregulated. After UAOS-Na treatment, 16 DEPs were enriched with 15 DEPs downregulated compared with EAE group, and these DEPs were markedly enriched in phagosome, MAPK, autoimmune thyroid disease, and antigen processing and presentation (APP) signaling pathway, etc. (Figure 6D). Among them, there were few studies on APP signaling pathway, so we focus on it. Meanwhile, GO functional annotation analysis of DEPs in UAOS-Na vs. EAE group showed that the results were closely related to APP signaling pathway (Figure 6E). DEPs were enriched in vesicle membranes and participate in biological processes such as antigen processing and presentation, membrane localization and so on. And in the APP signaling pathway, DEPs in the MHC-I antigen presentation pathway were significantly up-regulated in EAE, while DEPs such as H2-T23 was significantly down-regulated after UAOS-Na treatment (Figure 6F). MHC-I antigen presentation pathway is related to the uptake and processing of antigens and the activation and proliferation of CD8 T cells. Overall, downregulation of H2-T23, the key proteins in MHC-I antigen presentation pathway, may be an important molecular mechanism of UAOS-Na in the treatment of EAE.

### UAOS-Na suppresses MHC-I expression and CD8 T cell infiltration in the CNS by inhibiting Tapbp and H2-T23 expression *in vivo*

**Figure 7:**
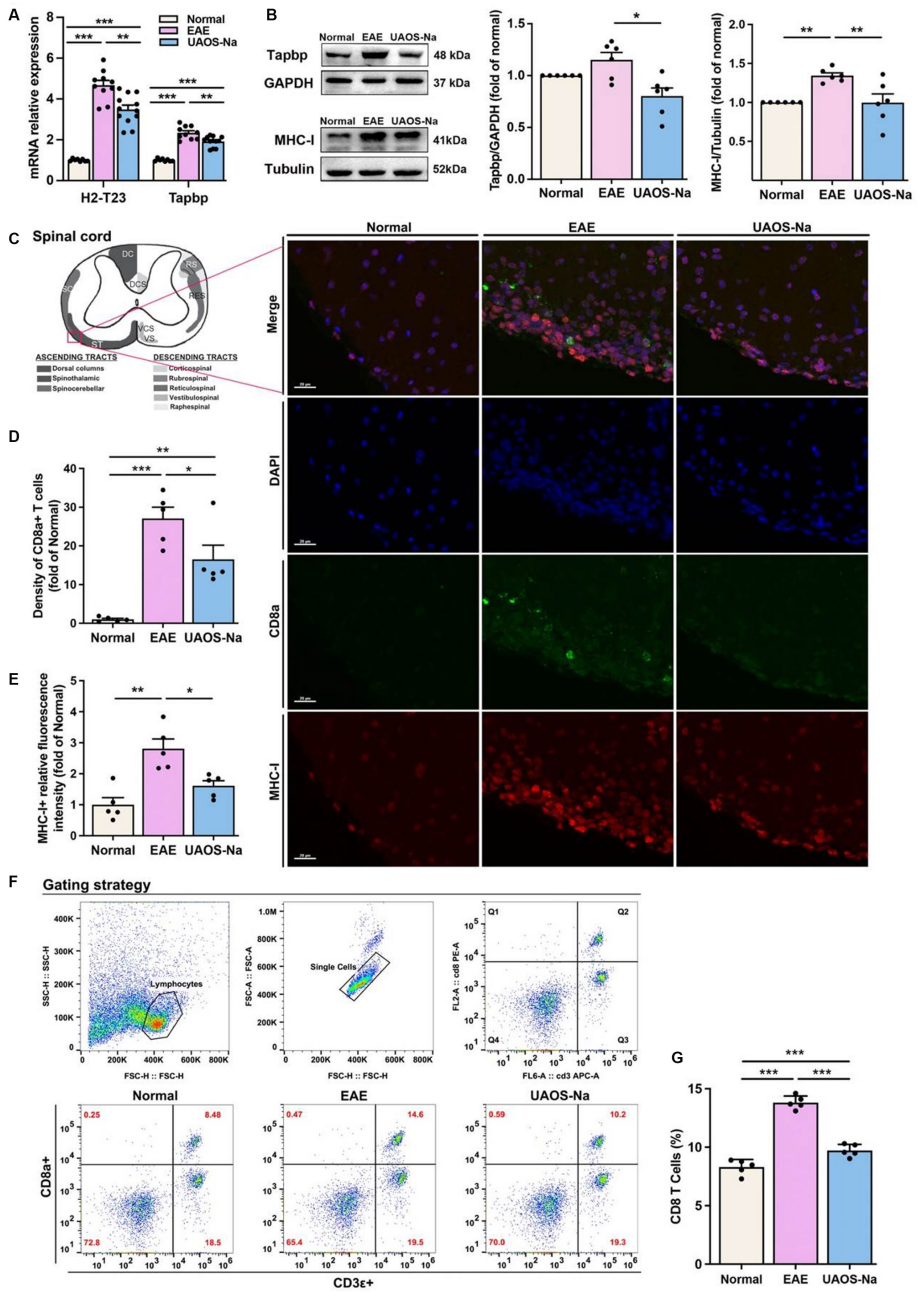
UAOS-Na reduces the MHC-I expression and CD8 T cell infiltration by inhibiting Tapbp and H2-T23 expression *in vivo*. Female, 6 to 8 weeks-old C57BL/6J mice were immunized with MOG35-55 and treated with UAOS-Na (60 mg/kg/d) by oral gavage from onset to 25 dpi. **(A)** mRNA levels of *H2-T23* and *Tapbp* in mouse spinal cord analyzed by real-time PCR (*n* = 8 to 12). **(B)** The protein bands and the relative expression levels of Tapbp and MHC-I of mouse spinal cord detected by western blot (*n* = 6, student’s *t*-test was performed). **(C)** Spinothalamic immunofluorescence staining of mouse spinal cord (scale bar = 20 μm). **(D,E)** Image-Pro analysis of CD8a^+^ T cell density and MHC-I fluorescence intensity (*n* = 5, student’s *t*-test was performed). **(F)** The levels of CD8a^+^ and CD3ε^+^ T cells in spleen were detected by flow cytometry. **(G)** The proportion of CD8 T cells in CD3 T cells (*n* = 5). Data were showed by mean ± SME, one-way ANOVA with Tukey’s multiple comparisons test was performed. ^*^p < 0.05, ^**^p < 0.01, and ^***^p < 0.001.

It has been shown that the TAP-related protein (Tapbp), plays a crucial role in MHC-I antigen presentation pathway. Tapbp is involved in stabilizing MHC-I molecules (41, 42), and increasing the assembly speed and number of MHC-I (43–45). Therefore, both H2-T23 and Tapbp were investigated in subsequent experiments. As shown in Figure 7A, *Tapbp* and *H2-T23* mRNA expression levels in mouse spinal cord were assayed by Real-Time PCR. The results showed that *Tapbp* and *H2-T23* mRNA levels were significantly increased in EAE group compared with normal group. However, UAOS-Na treatment markedly reduced these mRNA expression levels.

Previous studies have reported that restriction of Tapbp and H2-T23 expression resulted in decreased MHC-I expression on the cell membrane and inhibited CD8 T cell activation (46, 47). To analyze Tapbp and MHC-I protein expression levels in spinal cord tissues, western blotting was performed. The results revealed that Tapbp and MHC-I protein expression levels in EAE mouse spinal cords were higher than those in normal mouse spinal cords, and those protein expression levels were obviously decreased after UAOS-Na treatment (Figure 7B). In addition, to examine whether UAOS-Na treatment could reduce the CNS infiltration of CD8 T cells and the expression of MHC-I in EAE mouse spinal cord, we analyzed the MHC-I expression and the density of CD8^+^ T cells in mouse lumbar spinal cord by immunofluorescence staining. As shown in Figure 7C, compared with normal group, the density of CD8^+^ T cells and high MHC-I expression cells were higher in EAE group. UAOS-Na treatment reversed the results compared with EAE group (Figures 7D,E). Thus, UAOS-Na could inhibit the MHC-I expression and reduce the CD8 T cell infiltration in EAE mouse spinal cords. Furthermore, the proportion of CD8^+^ T cells in mouse spleen was detected by flow cytometry, and the results showed that UAOS-Na significantly reduced the proportion of CD8 T cells (CD3ε^+^, CD8a^+^) in splenocytes of EAE mice (Figures 7F,G). Overall, UAOS-Na reduced MHC-I expression in the spinal cord by inhibiting Tapbp and H2-T23 and reduced peripheral proportion and CNS infiltration of CD8 T cells.

### UAOS-Na reduces the antigen presentation ability of mBMDC by inhibiting Tapbp and H2-T23 expression *in vitro*

**Figure 8:**
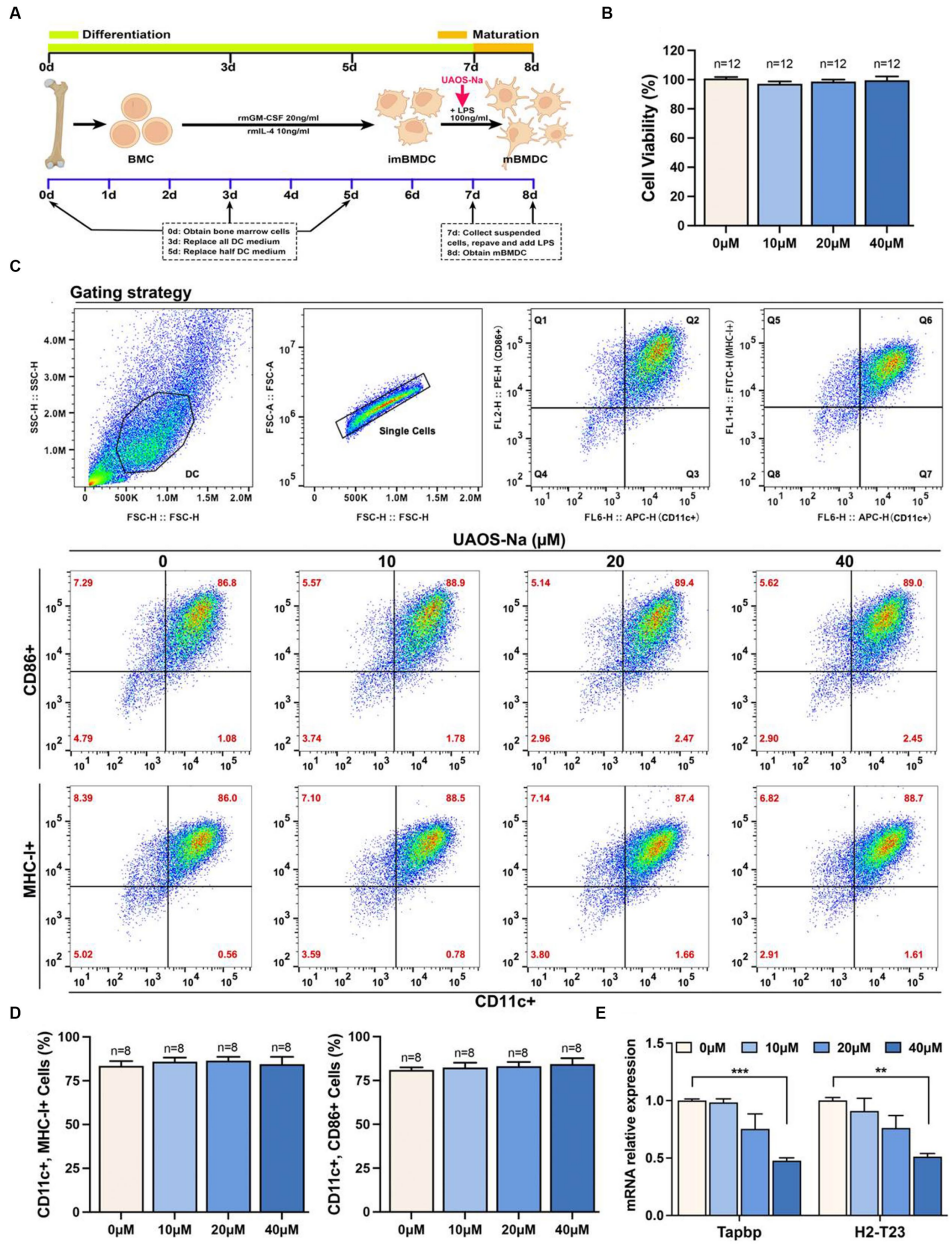
UAOS-Na reduces the antigen presentation ability of mBMDC. **(A)** Schematic diagram of mBMDC induction. **(B)** Cell viability was analyzed by CCK-8 commercial kits. **(C)** Flow cytometry was used to detect the levels of CD11c, CD86 and MHC-I. **(D)** The proportion of mBMDC. **(E)** mRNA levels of *Tapbp* and *H2-T23* in mBMDC. Data were showed as mean ± SME. Compared with UAOS-Na-0 μM group, one-way ANOVA with Tukey’s multiple comparisons test was performed, ^**^p < 0.01 and ^***^p < 0.001.

We investigated the effect of UAOS-Na on mBMDC, an *in vitro* model of antigen-presenting cells, induced by C57BL/6 mouse bone marrow cells (Figure 8A). CCK-8 results showed that UAOS-Na did not affect the viability of mBMDC (Figure 8B). To evaluate the effect of UAOS-Na on the BMDC maturation, flow cytometry was performed. UAOS-Na did not affect the numbers of mBMDC (CD11c^+^, CD86^+^ and CD11c^+^, MHC-I^+^) (Figures 8C,D). However, UAOS-Na reduced the mRNA levels of *Tapbp* and *H2-T23* in mBMDC (Figure 8E), which indicated that UAOS-Na could reduce the antigen presentation ability of mBMDC.

**Figure 9:**
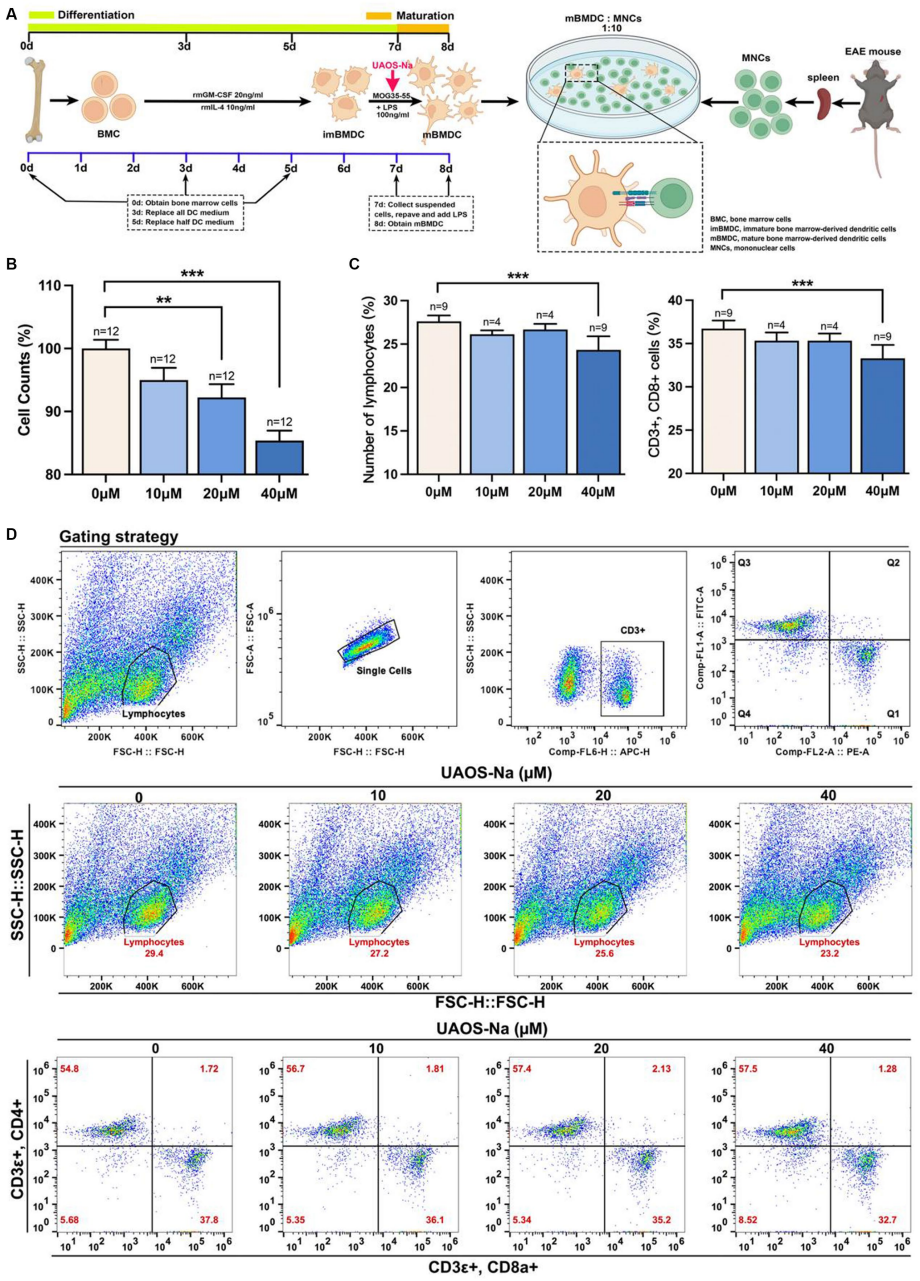
UAOS-Na reduces the proliferation and activation of CD8 T cells *in vitro*. **(A)** Schematic diagram of MOG-mBMDC activating MOG-T cells. **(B)** CCK-8 commercial kits were used to detect the inhibitory effect of UAOS-Na on MOG-T cell proliferation induced by MOG-mBMDC. **(C,D)** Flow cytometry was used to detect the levels of CD3ε, CD8a and CD4 **(D)**, and the proportion of lymphocytes and CD8 T cells (**C**, *n* = 4–9 per group). Data were showed as mean ± SME. one-way ANOVA with Tukey’s multiple comparisons test was performed, ^*^p < 0.05, ^**^p < 0.01, and ^***^p < 0.001.

To investigate the effect of UAOS-Na on the antigen presentation ability of mBMDC, MOG-mBMDC treated by UAOS-Na were co-cultured with MOG-lymphocytes derived from EAE mouse spleen (Figure 9A). As shown in Figure 9B, UAOS-Na treatment inhibited MOG-lymphocytes proliferation induced by MOG-mBMDC which was analyzed by CCK-8 commercial kits. Furthermore, in Figures 9C,D, flow cytometry was used to determine the effect of UAOS-Na on the proportion of lymphocytes and CD8 T cells (CD3ε^+^, CD8a^+^) in the co-cultured system. UAOS-Na (40 μM) significantly inhibited the anumber of lymphocytes and CD8 T cells (CD3ε^+^, CD8a^+^; Figures 9B–D).

In conclusion, UAOS-Na inhibited mBMDC antigen presentation ability by reduced Tapbp and H2-T23 expression thereby suppressing CD8 T cell proliferation and differentiation.

## Discussion

Given the inflammation and demyelination lesions in the acute stage of MS/EAE, an ideal therapeutic strategy would have the capacity both to inhibit the inflammation and to reduce the axon injury with minimal side effects. Previous studies have reported that UA has immunomodulation and direct anti-demyelination effects on EAE (21–24). However, UA is insoluble in water, which may affect its medication effectiveness. To promote the aqueous solubility of UA, UAOS-Na, a derivative of UA, was prepared in our previous study. In the present study, UAOS-Na was used to treat EAE, and the results showed that UAOS-Na delayed the onset time, reduced the incidence, ameliorated inflammation and astrogliosis, and protected myelin sheath in MS animal models. Mechanistically, UAOS-Na treatment downregulated the protein levels of Tapbp and H2-T23 in MHC-I antigen presentation pathway and reduced the proliferation of splenic CD8 T cells, thereby reduced the CNS infiltration of CD8 T cells and CNS injury.

Literatures have reported that the imbalance of serum pro-inflammatory and anti-inflammatory cytokines is closely related to EAE inflammation, and inhibiting the expression of pro-inflammatory cytokines such as IFN-γ, TNF-α, IL-6, and IL-17 (31, 32) and promoting the expression of anti-inflammatory cytokines such as IL-10 (33, 34) can alleviate the inflammation of EAE. And small molecules targeting T-bet and RORγt also inhibit EAE (35). UA could reduce the serum levels of IL-6, IFN-γ, TNF-α and IL-17 (22, 24, 48). In the present study, UAOS-Na-treated EAE mice showed a significant reduction in serum levels of IFN-γ, TNF-α, IL-6, and IL-17A and in expression of key transcription factors T-bet and ROR-γt in spinal cord. In parallel, H&E staining of the spinal cord visually demonstrated that UAOS-Na significantly reduced the CNS inflammation in EAE mice. These results suggested that UAOS-Na reduced peripheral and CNS inflammation in EAE mice (Figures 2, 3). In addition, UA distribution depended on the blood flow and perfusion rate of the organs as follows: lung > spleen > liver > cerebrum > heart > kidney (49), and UA can treat central nervous system diseases (16, 17, 24). In our previous study, the *in vivo* pharmacokinetic parameters of UAOS-Na and UA indicated that UAOS-Na was 6-fold more exposed than UA, and that UAOS-Na was biologically active *in vivo* as a prodrug of UA (these data are not shown in this paper). These data also support that UAOS-Na has therapeutic activity of CNS inflammation and may be more effective than UA.

In addition to inflammation, the main pathology of MS includes demyelination and axonal injury (50, 51). Axonal injury after demyelination in MS is difficult to reverse and is a key component of disease progression and permanent neurologic disability. Therefore, preventing demyelination and promoting remyelination is very important and has become one of the research difficulties and hotspots in MS. (52) In this study, H&E (inflammation) and LFB (demyelination) staining of EAE mice spinal cord were performed and the results showed that UAOS-Na had anti-inflammatory and myelin protective effects (Figure 2). However, the effect of UAOS-Na on demyelination in EAE mice may be secondary to its immunomodulatory effect. To test whether UAOS-Na has a direct effect on myelin, we used the cuprizone-induced demyelination mouse model with minimal inflammation and highly effective remyelination. Cuprizone mice were treated with UAOS-Na from weeks 2 to 6, and the results suggested that UAOS-Na may have a direct protective effect on myelin (Figure 4), which is consistent with the anti-demyelinating effect of UA mentioned earlier. In the cuprizone model, however, demyelination and remyelination occur simultaneously in the corpus callosum from weeks 2 to 6. Therefore, whether UAOS-Na prevents demyelination or boost myelin regeneration needs to be verified by further experiments. In addition, cuprizone treatment also enhanced the reactivity of the central resident cells, such as astrocytes and microglia, which is accompanied by expression of pro-inflammatory cytokines and other inflammatory modulators (53). And astrocytes become hypertrophic and hyperplastic predates demyelination (54, 55). In this study, UAOS-Na ameliorates astrogliosis in EAE mice and cuprizone-induced mice (Figure 5), which may be a potential factor for the protection of myelin sheath by UAOS-Na. Furthermore, studies have shown that microglia become reactive as early as 1 week after cuprizone induction and sustain activity throughout demyelination (56, 57). However, whether UAOS-Na can protect the CPZ corpus callosum myelin by affecting microglia was not investigated.

For inflammatory mechanisms, MS inflammation begins with the uptake, processing, and presentation of myelin-specific antigens by antigen presenting cells (APCs) and subsequent T cell activation (58). In MHC-I antigen presentation pathway, antigens are degraded into short peptides and bound to the MHC-I antigen-binding groove in ER to form peptide-MHC-I complexes (pMHC-I), which are subsequently expressed on the cell surface for recognition by CD8 T cells (59). TCRs on CD8 T cells interact with pMHC-I, a signaling cascade is initiated resulting in cytokine production, proliferation, and/or cytolysis (47, 60). And CD8 T cells play an important role in MS and EAE pathology (61–64). Literatures have revealed that in a variety of neurological diseases, high MHC-I expression on neurons makes them a killing target of CD8 T cells, which eventually leads to neuronal apoptosis and synaptic junction dysplasia (65–68). In MS, neurons, AST, and OLs are the CD8 T cell killing targets because of their high MHC-I expression (69–71). Therefore, CD8 T cell mediated CNS damage dependent on MHC-I antigen presentation pathway may be one of the causes of MS or EAE lesions. In this study, proteomics results indicated that UAOS-Na downregulated the expression of H2-T23 in MHC-I antigen presentation pathway (Figure 6). H2-T23 forms non-classical MHC-Ib with β2m on the cell membrane, which can bind and present antigen, and participate in the positive regulation of CD8 T cell-mediated cytotoxicity (46, 72–74). Moreover, Tapbp, expressed in all nucleated cells, is essential for the successful surface expression of pMHC-I (75). Tapbp is involved in stabilizing MHC-I molecules (41, 42), and increasing the assembly speed and number of MHC-I (43–45). When Tapbp expression is restricted, pMHC-I expression in the cell membrane, the antigen presentation process, and CD8 T cell development are inhibited (47). Hence, we hypothesized that UAOS-Na relieved CNS inflammation in EAE mice, which was related to the inhibition of Tapbp and H2-T23 expression. And this hypothesis has been verified by *in vitro* and *in vivo* experiments. Further studies are needed to determine whether UAOS-Na protects myelin sheath in relation to its reduction of Tapbp expression in cells, especially oligodendrocytes. Furthermore, existing studies suggested that CD8 Treg cells play an important role in MS/EAE remission (76, 77), which was not discussed in depth in this paper. We will investigate different subtypes of CD8 T cells in the future. Interactions of NK cell receptors with pMHC-I on cell surfaces plays a crucial role in the licensing or education of NK cells (78); and microglia considered to be the principal APC within the CNS may modulate the CD8 T cells response including their proliferation, differentiation and apoptosis through the antigen presenting mechanism (79, 80); however, whether UAOS-Na can affect the function of NK cells and microglia by reducing the cell expression of Tapbp and MHC-I needs further study.

During our experiments, all animals were weighed, scored, and treated at a fixed time each day. Mice were given individualized treatment, that is, the mice were treated only after they developed clinical symptoms (onset). In our study, most of the mice had a consistent onset, which manifested as decrease in tail tension with score point 1 (83.3%) and completed tail paralysis with score point 2 (13.5%). Therefore, we did not administer different doses according to disease severity. In addition, the onset of modeling mice had a subtle difference, which were mainly concentrated in 10–14 dpi. Meanwhile, in our preliminary study for EAE modeling (the data did not show in the paper), we found the demyelinating state was most serious in 25dpi and inflammatory state was recovered quickly after 25 dpi in the mouse spinal cord. In this study, combined consideration of demyelinating and inflammatory states, we chose 25 dpi as the experimental endpoint.

**Figure 10:**
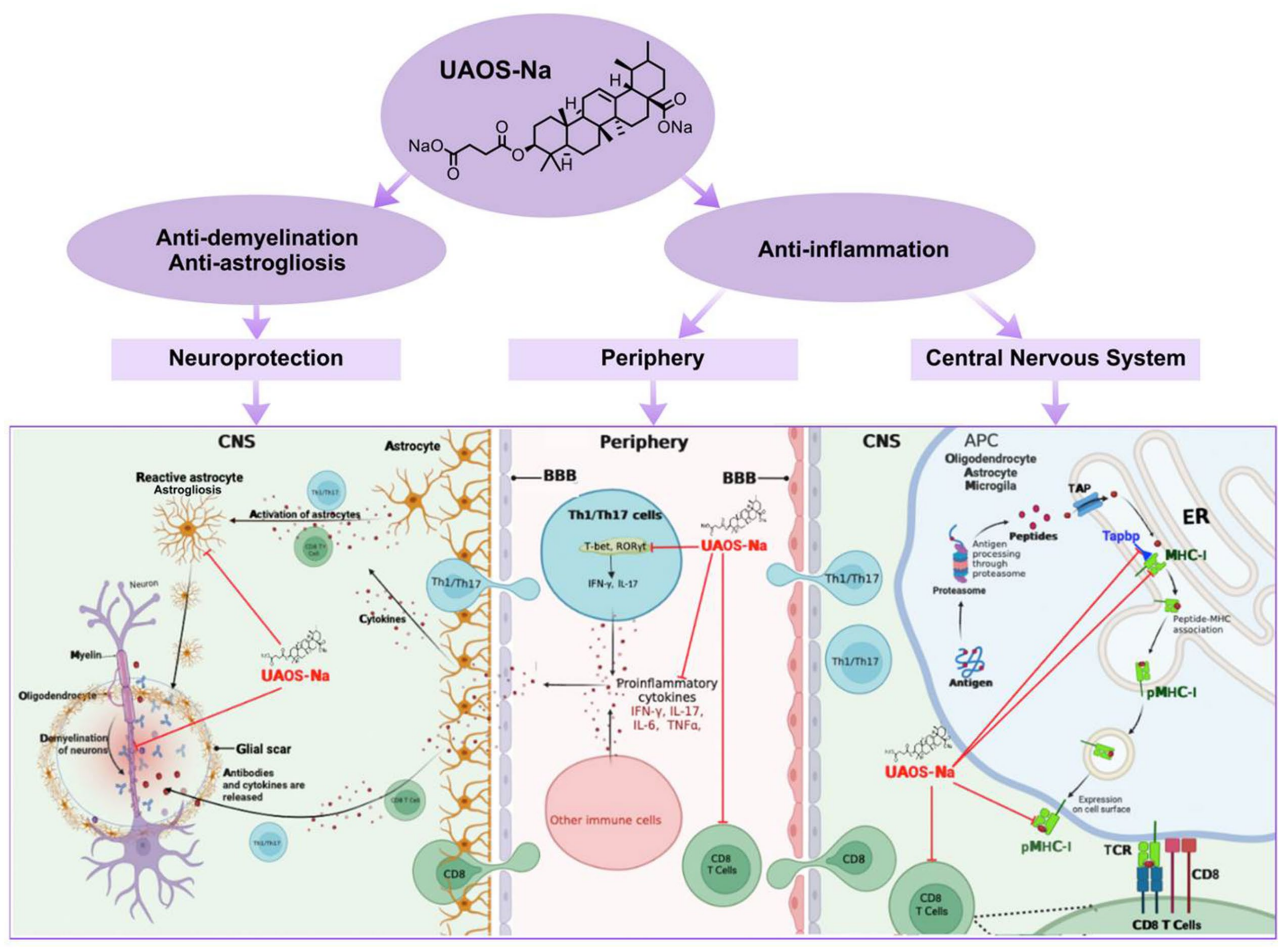
UAOS-Na-mediated immunomodulation and neuroprotection in EAE mice. In the periphery of EAE, UAOS-Na suppressed inflammation by decreasing serum proinflammatory factor levels of IFN-γ, IL-17A, TNF-α and IL-6 and inhibiting CD8 T cell activation (middle). In the CNS, UAOS-Na reduced the expression levels of Tapbp, H2-T23, and MHC-I and reduced CD8 T cell infiltration, thereby reducing the targeted killing of nerve cells by CD8 T cells (right). Moreover, UAOS-Na exerted neuroprotective effect by suppressing astrogliosis and protecting myelin sheath (left).

Summary, in the periphery of EAE, UAOS-Na suppressed inflammation by decreasing serum proinflammatory factor levels of IFN-γ, IL-17A, TNF-α and IL-6 and inhibiting CD8 T cell activation. In the CNS of EAE, UAOS-Na reduced the expression levels of Tapbp, H2-T23, and MHC-I and reduced CD8 T cell infiltration, thereby reducing the targeted killing of nerve cells by CD8 T cells. Moreover, UAOS-Na exerted neuroprotective effect by suppressing astrogliosis and protecting myelin sheath (Figure 10).

## Data availability statement

The original contributions presented in the study are publicly available. This data can be found at: [ProteomeXchange/PXD045108].

## Ethics statement

The animal study was approved by Ethics Committee of Animal Experiments of Shanghai University of Traditional Chinese Medicine. The study was conducted in accordance with the local legislation and institutional requirements.

## Author contributions

MW: Conceptualization, Data curation, Investigation, Methodology, Software, Visualization, Writing – original draft. CG: Data curation, Methodology, Visualization, Writing – review & editing. YY: Formal analysis, Investigation, Supervision, Writing – review & editing. LC: Investigation, Methodology, Validation, Writing – review & editing. KC: Project administration, Resources, Writing – review & editing. JD: Investigation, Methodology, Supervision, Validation, Writing – review & editing. HW: Data curation, Writing – review & editing, Conceptualization, Investigation. YL: Conceptualization, Methodology, Project administration, Resources, Writing – review & editing.

## Footnotes

^1^https://david.ncifcrf.gov

## Glossary

APC: Antigen presenting cell
APP: Antigen processing and presentation
AST: Astrocyte
BBB: Blood brain barrier
BMDC: Bone marrow-derived dendritic cells
BP: Biological process
CC: Cellular component
CMC-Na: Sodium carboxymethylcellulose
CNS: Central nervous system
CPZ: Cuprizone
Ct: Cycle threshold
DCs: Dendritic cells
DEPs: Differential expression proteins
dpi: Days post immunization
EAE: Experimental autoimmune encephalomyelitis
ELISA: Enzyme-linked immunosorbent assay
ER: Endoplasmic reticulum
FC: Fold change
GFAP: Glial fibrillary acidic protein
GO: Gene Ontology
H2-T23: H-2 class I histocompatibility antigen D-37 alpha chain, H2-T23
IFN-γ: Interferon γ
KEGG: Kyoto Encyclopedia of Genes and Genomes
IL-10: Interleukin 10
IL-17: Interleukin 17
IL-2: Interleukin 2
IL-6: Interleukin 6
imBMDC: Immature bone marrow-derived dendritic cells
mBMDC: Maturate bone marrow-derived dendritic cells
MF: Molecular function
MHC-I: Major histocompatibility complex class I
MNCs: Mononuclear cells
MOG: Myelin oligodendrocyte glycoprotein
MS: Multiple sclerosis
OLs: Oligodendrocyte
OPCs: Oligodendrocyte precursor cells
PBS: Phosphate buffered saline
PFA: Paraformaldehyde
pMHC-I: Peptide-MHC-I complexes
RORγt: RAR-related orphan receptor gamma
SI: Spleen index
TAP: Transporter associated with antigen processing
Tapasin: TAP binding protein
Tapbp: TAP binding protein (house mouse)
T-bet: T-box transcription factor 21
Th: T helper cells
TMT: Tandem mass tag
TNF-α: Tumor necrosis factor α
UA: Ursolic acid
UAOS-Na: Ursolic acid disodium succinate monoester
